# Supplementary material for: A single mutation G454A in the P450 CYP9K1 drives pyrethroid resistance in the major malaria vector Anopheles funestus reducing bed net efficacy
Source: Genetics. 2024 Nov 7;229(1):iyae181. doi: 10.1093/genetics/iyae181 (PMC11708915; doi:10.1093/genetics/iyae181)
Supplement: iyae181_Supplementary_Data [file iyae181_supplementary_data.zip › Supplemental_Material_GENETICS-2024-307544.docx]

**Supplemental Material**

**1. Polymorphism analysis of *CYP9K1* gene across Africa**

Polymorphisms in 1614 bp coding fragments spanning the full *CYP9K1* gene were analyzed between permethrin-resistant female *An. funestus* mosquitoes from each of the three countries (Uganda, Cameroon and Malawi) and laboratory susceptible and resistant strains FANG and FUMOZ, respectively. The *CYP9K1* genetic diversity were retrieved from the SNP multi-sample report file generated through Strand NGS 3.4 for each population. Bioedit (Hall, 1999) was used to input various polymorphisms in the vector base reference sequence using ambiguous letter to indicate heterozygote positions.

Secondly, the full-length cDNA fragments of this gene were amplified from permethrin-resistant *An. funestus* mosquitoes collected in 2020 from Mayuge, Uganda, in 2020 from Mibellon, Cameroon, from Obuasi, Ghana in 2020, and from Chikwawa, Malawi in 2020. Full-length cDNA of *CYP9K1* was also amplified from FANG and FUMOZ. Amplifications were carried out using cDNA prepared from10 mosquitoes, using the Phusion high fidelity DNA polymerase (Thermo Ficher Scientific Waltham, Massachusetts, USA), using the primers provided in Table S2. The 15µl PCR mix comprised 3µl of 5x HF buffer (containing 1.5 mM MgCl_2_), 0.12µl of 25mM dNTPs, 0.51µl of 10mM forward and reverse primers, 0.15µl Phusion Taq, 9.71µl of deionised water and 1µl of DNA. Thermocycling conditions were one cycle at 98°C for 1 min; 35 cycles each of 98°C for 20 s (denaturation), 60°C for 30 s (annealing), and extension at 72°C for 2 min; and one cycle at 72°C for 10 mins (final elongation). PCR products were gel-purified using the QIAquick Gel Extraction Kit (Qiagen, Hilden, Germany), and ligated into pJET1.2/blunt cloning vector using the CloneJET PCR Cloning Kit (Thermo Ficher Scientific Waltham, Massachusetts, USA). The recombinant *CYP9K1-pJET1.2* were used to transform cloned *E*. *coli DH5α* cells, recombinant plasmids miniprepped using the QIAprep Spin Miniprep Kit (Qiagen, Hilden, Germany) and sequenced on both strands using pJET1.2-specific primers (Microsynth AG, Switzerland) (Table S2).

**2. Assessment of the association between the G454A-*CYP9K1* mutation and insecticide resistance through *in vivo* experiments**

The full-length *CYP9K1* was amplified from miniprep templates of the dominant alleles from 1. above, using Phusion high fidelity DNA polymerase (Thermo Ficher Scientific Waltham, Massachusetts, USA) and primers bearing EcoRI and *Xba*I restriction sites (Table S2). The PCR products were gel-purified using the QIAquick Gel Extraction Kit (Qiagen, Hilden, Germany), ligated into pJET1.2/blunt cloning vector (Thermo Ficher Scientific Waltham, Massachusetts, USA) and cloned. Minipreps were then digested from the pJET1.2/blunt vector using *EcoR*I and *Xba*I, purified and cloned in the *pUASattB* expression vector. These constructs were used to inject germ line transgenic flies by Cambridge Fly facility. Details of this protocol and balancing of flies were provided in previous studies (Riveron et al. 2013; Riveron et al. 2014; Ibrahim et al. 2015) The primers used are listed in Supplementary Table S2. Ubiquitous expression of *CYP9K1* candidate alleles in the transgenes in adult F_1_ progenies (experimental group) was achieved after crossing homozygote males (either 454A*-CYP9K1-UAS-UGA* or G454*-CYP9K1-UAS-FANG* UAS lines) with virgin females from the driver strain Actin5C-GAL4. For control group, flies with the same genetic background as the experimental group but devoid of the gene of interest (pUASattb-*CYP9K1* insertion) were crossed with the driver Actin5C-GAL4 lines to generate null-Actin5C-GAL4 lines. All flies were maintained at 25^o^C in plastic vials with food.

***3. Crossing between field and laboratory mosquito strains***

To segregate the various *CYP9K1* genotypes, two reciprocal crosses were carried out. The first was made between the female FANG vs field male resistant strains *An.* *funestus* from Mibellon, Cameroon (FANG x Mibellon) and the second crossing was made between FANG and the male resistant field strain from Mayuge, Uganda (FANG x Mayuge). 100 F_1_ adult male *An. funestus* mosquitoes from Mayuge and from Mibellon were separately crossed with 120 females FANG. The crossings were followed up to F_3_ generation for FANG x Mibellon and the F_5_ generation for FANG x Mayuge. These hybrid progenies were used to investigate the correlation between 454A-*CYP9K1* mutation and pyrethroid resistance

**4. Design of simple DNA-based diagnostics around the G454A-*CYP9K1* mutation**

***AS-PCR Genotyping protocol for the G454A-CYP9K1 mutation***

The genotyping was carried out using 10 mM of each of the primers listed in Table S3, and 1µl of genomic DNA in 15 µl reaction mix comprise of 10X Kapa Taq buffer A, 0.2 mM dNTPs, 1.5 mM MgCl2, 1U Kapa Taq (Kapa Biosystems, Wilmington, MA USA). The thermocycling parameters were: 1 cycle at 95 °C for 2 min; 30 cycles of 94 °C for 30 s, 59 °C for 30 s, 72 °C for 1 min and then a final extension step at 72 °C for 10 min. PCR products were separated on 1.5% agarose gel electrophoresis, stained with Midori Green advance DNA Stain (Nippon Genetics Europe GmbH) and visualised on a UV transilluminator.

***G454A-CYP9K1 locked nucleic acid (LNA) Protocol***

Each LNA assay comprised of a final concentration of 1× PrimeTime Master Mix (Integrated DNA technologies, UK) or 1× Luna Universal qPCR Master Mix (NEB), 0.1 µM for each of the two probes (LNA9K1-Gly: Hex and LNA9K1-Ala: Fam), 0.2 µM of primers (LNA-9K1F and LNA-9K1R) in a total reaction volume of 10 µl, containing 1 µl of DNA template. Reactions were set up in optical PCR tubes and run on an AriaMX Real-Time qPCR cycler (Agilent, USA) with Fam and Hex filters. The thermal cycling conditions were 3 mins denaturation at 95°C; 20 cycles of denaturation for 15 s at 95°C, annealing for 30 s at 66°C; 23 cycles of denaturation for 10 s at 95°C, annealing for 20 s at 58°C, and an extension for 10 s at 72°C.

**References**

Hall, 1999. Hall, T.A., 1999. BioEdit: a User-Friendly Biological Sequence Alignment Editor and Analysis Program for Windows 95/98/NT. In: Paper Presented at the Nucleic Acids Symposium Series - Google Search. [accessed 2023 Jun 4]. https://scholar.google.com/citations?view_op=view_citation&hl=en&user=25lPJlMAAAAJ&citation_for_view=25lPJlMAAAAJ:b0M2c_1WBrUC.

Ibrahim SS, Riveron JM, Bibby J, Irving H, Yunta C, Paine MJI, Wondji CS. 2015. Allelic Variation of Cytochrome P450s Drives Resistance to Bednet Insecticides in a Major Malaria Vector. PLoS Genet. 11(10):e1005618. doi:10.1371/journal.pgen.1005618.

Riveron JM, Irving H, Ndula M, Barnes KG, Ibrahim SS, Paine MJI, Wondji CS. 2013. Directionally selected cytochrome P450 alleles are driving the spread of pyrethroid resistance in the major malaria vector Anopheles funestus. Proc Natl Acad Sci U S A. 110(1):252–257. doi:10.1073/pnas.1216705110.

Riveron JM, Yunta C, Ibrahim SS, Djouaka R, Irving H, Menze BD, Ismail HM, Hemingway J, Ranson H, Albert A, et al. 2014. A single mutation in the GSTe2 gene allows tracking of metabolically based insecticide resistance in a major malaria vector. Genome Biol. 15(2):R27. doi:10.1186/gb-2014-15-2-r27.
