# Supplementary material for: A single mutation G454A in the P450 CYP9K1 drives pyrethroid resistance in the major malaria vector Anopheles funestus reducing bed net efficacy
Source: Genetics. 2024 Nov 7;229(1):iyae181. doi: 10.1093/genetics/iyae181 (PMC11708915; doi:10.1093/genetics/iyae181)
Supplement: iyae181_Supplementary_Data [file iyae181_supplementary_data.zip › Table_S1_GENETICS-2024-307544.docx]

**Table S1: Key Resource Table**

| REAGENT or RESOURCE | | SOURCE | IDENTIFIER |
| --- | --- | --- | --- |
| Chemicals, peptides, and recombinant proteins | | | |
| \| Phusion HF DNA polymerase \|  \| Cat# 10024537 \| \| --- \| --- \| --- \| | | Thermo Fisher | Cat# 10024537 |
| SYBR Green JumpStart Taq Ready mix | | Sigma-Aldrich | Cat# 10024537 |
| Brilliant III Ultra-Fast SYBR Green QPCR Master Mix | | Agilent | Cat# 600882 |
| NutriFly premix food | | SLS | Cat# FLY1034 |
| RESOURCE | |  |  |
| *Drosophila melanogaster*, genotype:  [‘‘y1w67c23; P attP40 25C6,’’ ‘‘1;2’’] | | Cambridge University |  |
| *Drosophila melanogaster*, genotype:  Act5C-GAL4 strain ‘‘y[1] w[*]; P(Act5CGAL4-w)E1/CyO,’’’’1;2’’] | | Bloomington, IN, USA | Act5C-GAL4 |
| Critical commercial assays | | | |
| SuperScript III Reverse Transcriptase kit | | | |
| PicoPure™ RNA Isolation Kit | | Thermo Fisher Scientific | Cat# KIT0204 |
| QIAprep® Spin Miniprep Kit | | QIAGEN | Cat# 27106 |
| Library Efficiency DH5a Competent cells | | Invitrogen | Cat# 18263012 |
| CloneJET PCR Cloning Kit | | Thermo Fisher | Cat# K1231 |
| JM109 Competent cells | | Promega | Cat# L2005 |
| Experimental models: Organisms/strains | | | |
| FANG | | (Hunt *et al.* 2005) | Lizette Koekemoer  University of the Witwatersrand |
| FUMOZ | | (Hunt *et al.* 2005) | Lizette Koekemoer  University of the Witwatersrand |
| FANG X Uganda (Mayuge) | | Centre for Research in Infectious Diseases (CRID) | This Paper |
| FANG X Cameroon (Mibellon) | | Centre for Research in Infectious Diseases (CRID) | This Paper |
| Oligonucleotides sequences used during the study | | | |
| Supplementary Materials | |  | See Table S2 and S3 |
| Recombinant DNA | | | |
| pUASTattB40 Vector | | (Riveron *et al.* 2013) | Generated by Wondji’s Group at LSTM |
| Pcw ori+ vector | | (Riveron *et al.* 2013) | Gift from Mark Paine, LSTM |
| Cytochrome P450 variants | CRID | | This paper |
| Cytochrome P450 reductase (CPR) | LSTM | | N/A |
| Cytochrome b5 | LSTM | | N/A |
| Software | | | |
| MEGA X | (Kumar *et al.* 2018) | | [www.megasoftware.net](http://www.megasoftware.net) |
| DnaSP6 | (Rozas *et al.* 2003) | | <https://dnasp.software.informer.com/> |
| GraphPad Prism8.0.2 | GraphPad Software | | https://www.graphpad.com/ |
| BioEdit 7.0.5 | (Hall, 1999) | | <https://bioedit.software.informer.com/7.0/> |
