# Supplementary material for: A single mutation G454A in the P450 CYP9K1 drives pyrethroid resistance in the major malaria vector Anopheles funestus reducing bed net efficacy
Source: Genetics. 2024 Nov 7;229(1):iyae181. doi: 10.1093/genetics/iyae181 (PMC11708915; doi:10.1093/genetics/iyae181)
Supplement: iyae181_Supplementary_Data [file iyae181_supplementary_data.zip › Table_S2_GENETICS-2024-307544.docx]

**Table S2.** **Primers used for *CYP9K1* cDNA Amplification and Functional Validation**

| **Primer Name** | **Forward Oligonucleotide Sequence** | **Reverse Oligonucleotide Sequence** |
| --- | --- | --- |
| CYP9K1-full | ATGCTGGGTACGCTCGTTGC | TCAGCAAGATTCTAGATCTAG |
| OMPA+2F | GGAATTCCATATGAAAAAGACAGCTATCGCG |  |
| OMPA+2CYP9K1_F | GCAACGAGCGTACCCAGCATCGGAGCGGCCTGCGCTACGGTAGCGAA |  |
| CYP9K1_R |  | TCTAGAGAATTCTTACGCTTGACGCGGCTGG |
| CYP9K1_UAS_F | GGGTACCGAATTCATGCTGGGTACGCTGGTTGCC |  |
| CYP9K1_UAS_R |  | GGTACCTCTAGATTACGCTTGACGCGGCTGGT |
| CYP9K1_UAS_qPCR_F | CGTACGATACGGTCCGAGAT |  |
| CYP9K1_UAS_qPCR_R |  | CAGGTTGGTAAGCTCCTTGC |

Restriction sites are in different colours and underlined: Brown is *EcoR*I, Blue is *NdeI*, Red is *KpnI*, Purple is *Xba*I, green is *Acc65I*.
