# Supplementary material for: A single mutation G454A in the P450 CYP9K1 drives pyrethroid resistance in the major malaria vector Anopheles funestus reducing bed net efficacy
Source: Genetics. 2024 Nov 7;229(1):iyae181. doi: 10.1093/genetics/iyae181 (PMC11708915; doi:10.1093/genetics/iyae181)
Supplement: iyae181_Supplementary_Data [file iyae181_supplementary_data.zip › Table_S3_GENETICS-2024-307544.docx]

**Table S3.** **Primers and Probes used for G454A-*CYP9K1* marker Genotyping**

| **Name** | **Forward sequence** | **Reverse Sequence** |
| --- | --- | --- |
| 9K1_OF | ACTGGACCGATGATGATTTGAC |  |
| 9K1_OR |  | ATCCAGAAGCCCTTCTCTGC |
| 9K1_IF | GGATCGTTTCTGGCCGGAAGGTTGG**C** |  |
| 9K1_IR |  | TATCGATCGGT GTCGGGCTGTCCG CT**C** |
| LNA-9K1F | CGTGATCCGCAACTGTTTC |  |
| LNA-9K1R |  | GTAAGGATGGACGCGGTATC |
| LNA9K1-Gly (Hex) | TCCGG+T+C+CGAAC |  |
| LNA9K1-Ala (Fam) | TCCGG+T+G+CG+AA |  |

LNA bases in red colour separated from other bases with plus sign (+).
