# Supplementary material for: A single mutation G454A in the P450 CYP9K1 drives pyrethroid resistance in the major malaria vector Anopheles funestus reducing bed net efficacy
Source: Genetics. 2024 Nov 7;229(1):iyae181. doi: 10.1093/genetics/iyae181 (PMC11708915; doi:10.1093/genetics/iyae181)
Supplement: iyae181_Supplementary_Data [file iyae181_supplementary_data.zip › Table_S4_GENETICS-2024-307544.docx]

**Table S4. Correlation between G454A-*CYP9K1* mutation and pyrethroid resistance phenotype.** Determined by WHO tube bioassay using female *An. funestus* hybrid and field samples alive and dead to pyrethroid exposure.

| **Samples/Insecticide** | **Comparison** | **OR** | **P value** | **CI** |
| --- | --- | --- | --- | --- |
| **FANG X Mayuge F5**  **Permethrin** | RR vs SS | 567 | <0.0001 | 34.03-5831 |
|  | RR vs RS | 9.88 | 0.025 | 1.485-114.1 |
|  | RS vs SS | 57.38 | <0.001 | 7.47-617.6 |
| **FANG X Mibellon F3**  **Alphacypermethrin** | RR vs SS | 50 | <0.0001 | 5.23-566.4 |
|  | RR vs RS | 4.5 | 0.2473 | 0.7124-52.42 |
|  | RS vs SS | 11.11 | <0.0001 | 3.31-32.28 |
| **Elende F1 deltamethrin** | RR vs SS | 11.33 | 0.03 | 1.25-136.1 |
|  | RR vs RS | 5.18 | 0.213 | 0.678-62.19 |
|  | RS vs SS | 2.187 | 01417 | 0.78-6.05 |
| **Elende F1 permethrin** | RR vs SS | 12.14 | 0.0256 | 1.554-149 |
|  | RR vs RS | 3.5 | 0.381 | 0.476-43.81 |
|  | RS vs SS | 3.46 | 0.0342 | 1.208-10.17 |
| **Elende F1 Alphacypermethrin** | RR vs SS | 7.1 | 0.0006 | 2.2-22.64 |
|  | RR vs RS | 0.57 | 0.6638 | 0.137-2.573 |
|  | RS vs SS | 6 | <0.001 | 1.879-17 |

* OR: odd ratio, CI: confidence interval, RR: homozygote resistant genotype, RS: heterozygote genotype, and SS: homozygote susceptible genotype.
