# Supplementary material for: A single mutation G454A in the P450 CYP9K1 drives pyrethroid resistance in the major malaria vector Anopheles funestus reducing bed net efficacy
Source: Genetics. 2024 Nov 7;229(1):iyae181. doi: 10.1093/genetics/iyae181 (PMC11708915; doi:10.1093/genetics/iyae181)
Supplement: iyae181_Supplementary_Data [file iyae181_supplementary_data.zip › Table_S5_GENETICS-2024-307544.docx]

**Table S5. Summary statistics of the correlation between G454A-*CYP9K1* marker and efficacy of LLINs by WHO cone test and EHTs.**

| **Samples/Insecticide** | **Comparison** | **OR** | **P value** | **CI** |
| --- | --- | --- | --- | --- |
| **WHO cone bioassay**  **Royal sentry** | RR vs SS | 20 | 0.0023 | 2.57-230.3 |
|  | RR vs RS | 5.6 | 0.811 | 66.04 |
|  | RS vs SS | 3.571 | 0.0223 | 1.184-9.511 |
| **Olyset** | RR vs SS | 11.5 | 0.0236 | 1.48-140-0 |
|  | RR vs RS | 2.667 | 0.6328 | 0.366-34.89 |
|  | RS vs SS | 4.313 | 0.0142 | 1.436-12.79 |
| **Experimental Hut Trials (EHTs)**  **Royal sentry** | RR vs SS | 3.818 | 0.148 | 0.825-19.29 |
|  | RR vs RS | 3.83 | 0.1475 | 0.83-19.41 |
|  | RS vs SS | 0.99 | >0.99 | 0.444-2.229 |
| **PermaNet 3.0** | RR vs SS | 0.9231 | >0.99 | 0.215-4.707 |
|  | RR vs RS | 0.269 | 0.113 | 0.068-1.157 |
|  | RS vs SS | 3.432 | 0.0057 | 1.509-7.645 |

* OR: odd ratio, CI: confidence interval RR: homozygote resistant genotype, RS: heterozygote genotype, and SS: homozygote susceptible genotype.
