# Supplementary figures and images for: A single mutation G454A in the P450 CYP9K1 drives pyrethroid resistance in the major malaria vector Anopheles funestus reducing bed net efficacy
Source: Genetics. 2024 Nov 7;229(1):iyae181. doi: 10.1093/genetics/iyae181 (PMC11708915; doi:10.1093/genetics/iyae181)

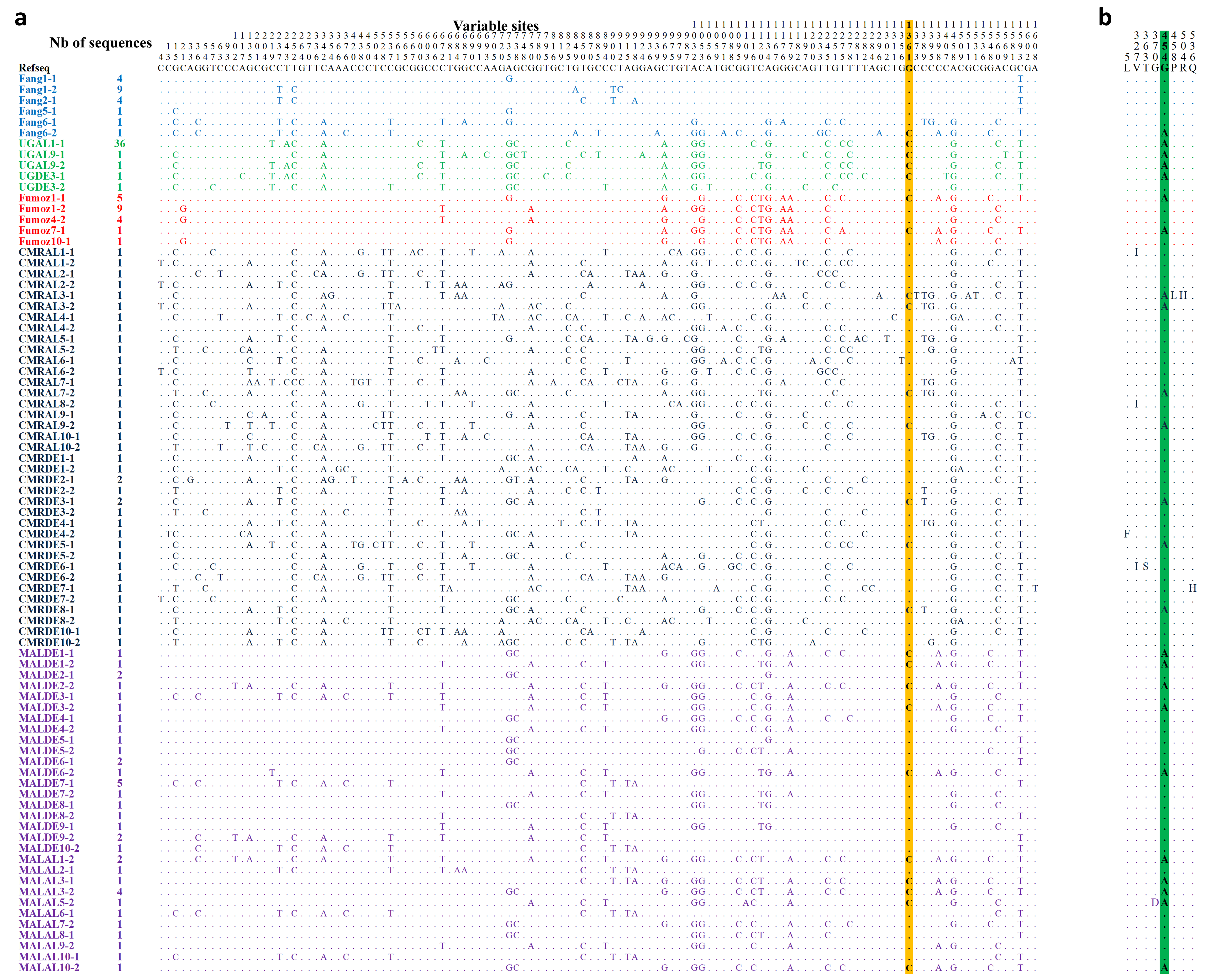

Supplement: iyae181_Supplementary_Data [file iyae181_supplementary_data.zip › Figure_S1_GENETICS-2024-307544.tif]

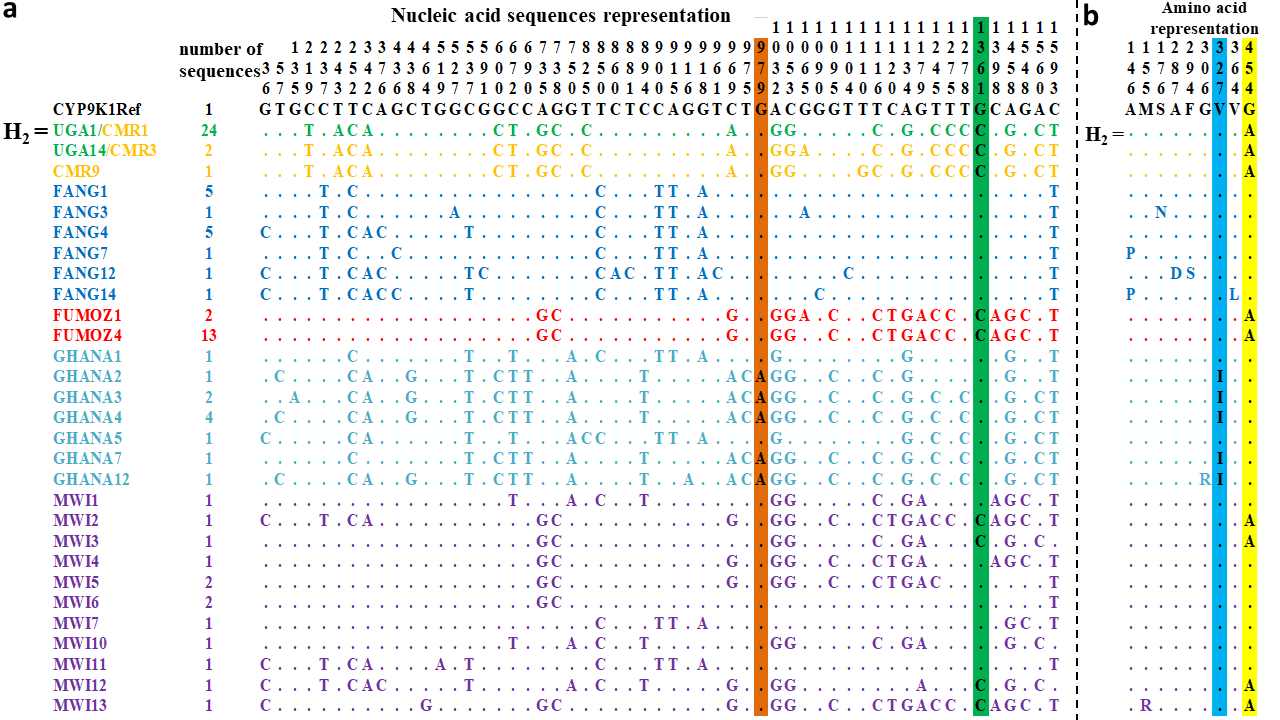

Supplement: iyae181_Supplementary_Data [file iyae181_supplementary_data.zip › Figure_S2_GENETICS-2024-307544.tif]

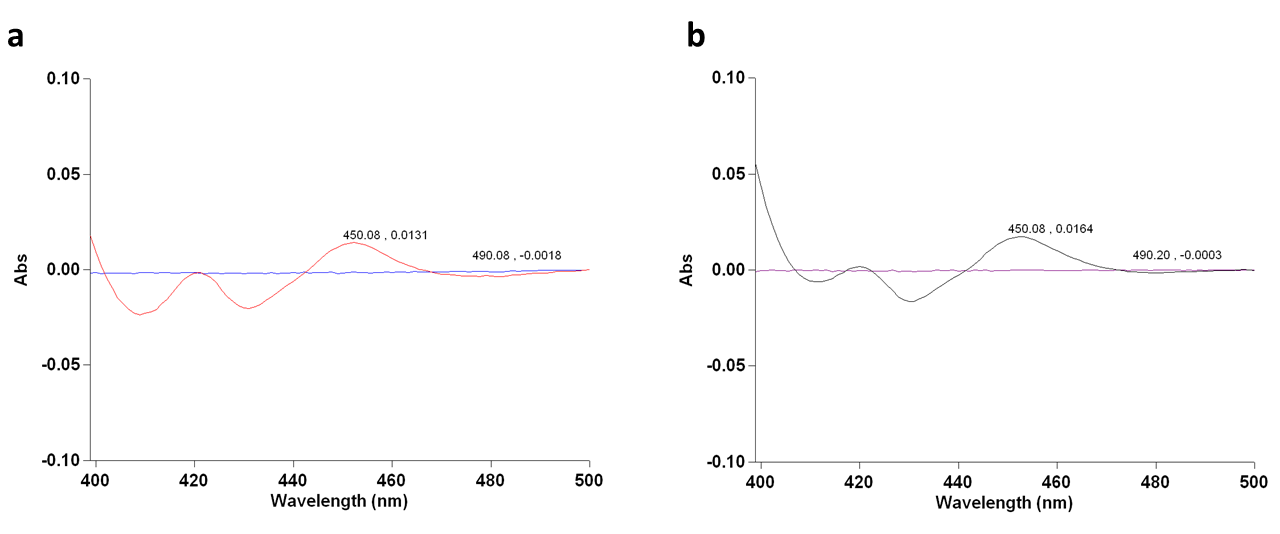

Supplement: iyae181_Supplementary_Data [file iyae181_supplementary_data.zip › Figure_S3_GENETICS-2024-307544.tif]

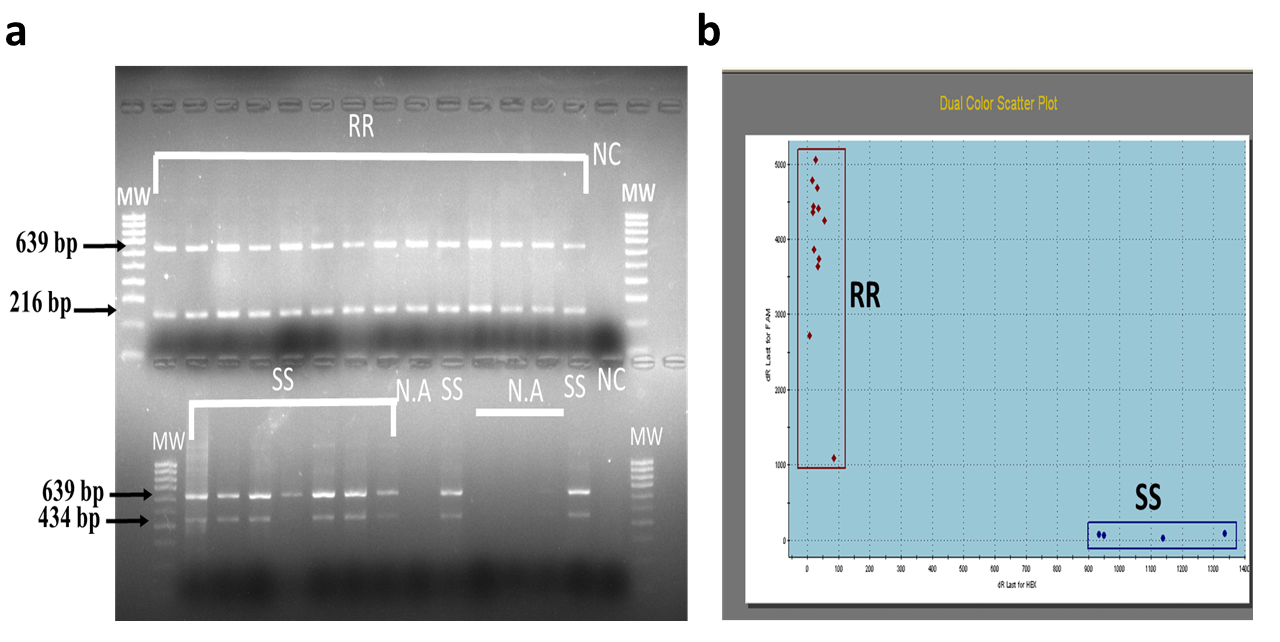

Supplement: iyae181_Supplementary_Data [file iyae181_supplementary_data.zip › Figure_S4_GENETICS-2024-307544.tif]

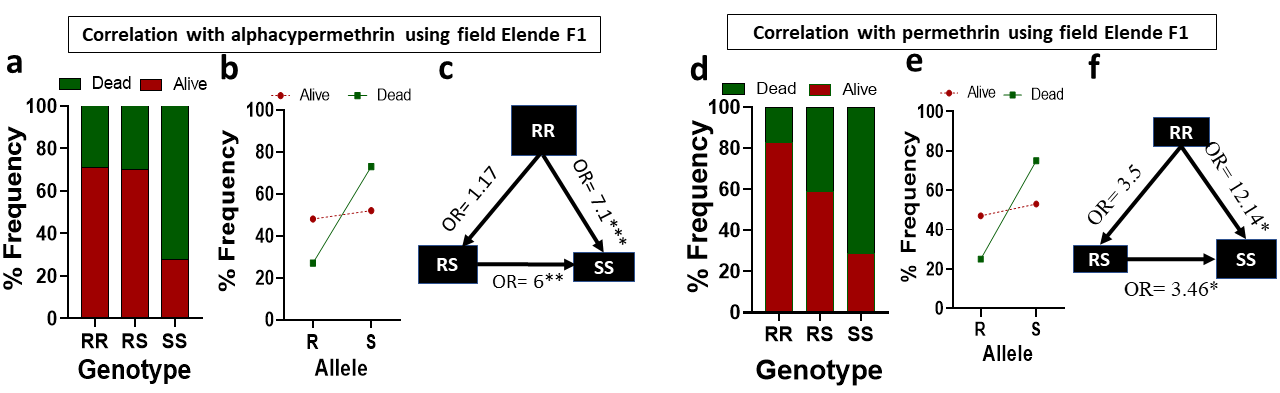

Supplement: iyae181_Supplementary_Data [file iyae181_supplementary_data.zip › Figure_S5_GENETICS-2024-307544.tif]
